# αDβ2 as a novel target of experimental polymicrobial sepsis

**DOI:** 10.3389/fimmu.2022.1059996

**Published:** 2022-11-18

**Authors:** Sophia Koutsogiannaki, Lifei Hou, Toshiaki Okuno, Miho Shibamura-Fujiogi, Hongbo R. Luo, Koichi Yuki

**Affiliations:** ^1^ Department of Anesthesiology, Critical Care and Pain Medicine, Cardiac Anesthesia Division, Boston Children’s Hospital, Boston, MA, United States; ^2^ Department of Anaesthesia, Harvard Medical School, Boston, MA, United States; ^3^ Department of Immunology, Harvard Medical School, Boston, MA, United States; ^4^ Department of Biochemistry, Juntendo University Faculty of Medicine, Tokyo, Japan; ^5^ Department of Pathology, Boston Children’s Hospital, Boston, MA, United States

**Keywords:** integrin, aDb2, sepsis, cell death, phagocytosis

## Abstract

Since sepsis was defined three decades ago, it has been a target of intensive study. However, there is no specific sepsis treatment available, with its high mortality and morbidity. αDβ2 (CD11d/CD18) is one of the four β2 integrin members. Its role in sepsis has been limitedly studied. Using an experimental polymicrobial sepsis model, we found that the deficiency of αDβ2 was associated with less lung injury and better outcome, which was in sharp contrast to other β2 integrin member αLβ2 (CD11a/CD18), and αMβ2 (CD11b/CD18). This phenotype was supported by a reduction of bacterial loads in αDβ2 knockout mice. Further analysis showed that the deficiency of αDβ2 led to a reduction of neutrophil cell death as well as an increase in neutrophil phagocytosis in both murine and human systems. Our data showed a unique role of αDβ2 among the β2 integrin members, which would serve as a potential target to improve the outcome of sepsis.

## Introduction

Sepsis remains to be a huge health care burden. Sepsis is the leading cause of death in the non-cardiac intensive care units (ICUs), accounting for more than 750,000 deaths annually in the U.S. Lack of specific treatment against sepsis is largely responsible for its high morbidity and mortality. Sepsis is treated conservatively with antibiotic administration, fluid resuscitation, and respiratory support. Thus, the development of a therapeutic to attenuate sepsis is urgently needed.

β2 integrins are a leukocyte-specific glycoprotein adhesion molecule family consisting of α- and β-subunits responsible for a number of leukocyte functions during infection. The critical role of β2 integrins in infection is well illustrated by a rare genetic disease called leukocyte adhesion deficiency type I (LAD I), which is caused by a functional or expressional defect of β2 integrins, and characterized by recurrent infection, sepsis, and death (1). β2 integrins consist of the four members; αLβ2 (CD11a/CD18, leukocyte function-associated antigen-1), αMβ2 (CD11b/CD18, macrophage 1-antigen), αXβ2 (CD11c/CD18), and αDβ2 (CD11d/CD18). Among them, αLβ2 and αMβ2 have been extensively studied. αLβ2 is ubiquitously expressed on leukocytes, and involved in a number of important leukocyte functions including trafficking and immunological synapse formation (2). αMβ2 is expressed mainly on innate immune cells, and plays a major role in their recruitment and phagocytosis (3, 4). Both αLβ2 and αMβ2 deficiency significantly worsened infection and sepsis outcome (5–7). αXβ2 (CD11c) is widely known as a dendritic cell marker, and its deficiency also worsened the sepsis outcome (8). αDβ2 was cloned last among the β2 integrins, and the investigation on the role of αDβ2 in sepsis has been limited.

The inhibition of β2 integrins as a whole may not be desirable based on LAD I phenotype. However, the blockade of β2 integrins mitigated lung injury in sepsis (9, 10), suggesting that a subset of the β2 integrins serves as a potential target for sepsis treatment. Here we hypothesized that the inhibition of a subset of β2 integrin members would serve to protect from lung injury and lead to the improvement of sepsis outcome. Accordingly, the aim of this study was to determine the role of each β2 integrin member in the development of sepsis-associated lung injury. We found that αDβ2 played a unique role in sepsis among the β2 integrin members so that its inhibition led to the improvement of sepsis outcome with less lung injury.

## Materials and methods

### Mice

Wild type (11), CD11a (αL) knockout (KO) mice (12), CD11b (αM) KO mice (13), CD11d (αD) KO mice (14) were obtained from Jackson Laboratory (Bar Harbor, Maine, USA). CD11c (αX) KO mice were kindly given by Dr. Christie Ballantyne (Baylor University). CD18 KO (β2) KO mice were kindly given by Dr. Dennis Wagner (Boston Children’s Hospital). They were housed under specific pathogen-free condition, with 12-hour light and dark cycles. All animal protocols were approved by the Institutional Animal Care and Use Committee (IACUC) at Boston Children’s Hospital.

### CLP surgery

All the experimental procedures complied with institutional and federal guidelines regarding the use of animals in research. Polymicrobial abdominal sepsis was induced by cecal ligation and puncture surgery, as we previously performed (7). In brief, mice were anesthetized with an intraperitoneal injection of ketamine 60 mg/kg and xylazine 5 mg/kg. Following its exteriorization, the cecum was ligated at a 1.0 cm from its tip and subjected to a single, through- and -through puncture using a 20-gauge needle. A small amount of fecal material was expelled with a gentle pressure to maintain the patency of puncture sites. The cecum was reinserted into the abdominal cavity. 0.1 mL/g of warmed saline was administered subcutaneously. Buprenorphine was given subcutaneously to alleviate postoperative surgical pain. For outcome study, mice were observed up to 7 days.

### Lung injury analysis

For histological analysis, lung was fixed with 4% paraformaldehyde for histological analysis. Lung histology was subjected to Hematoxylin and Eosin (H&E) staining. In some cases, we also examined bronchial lavage fluid (BAL) for protein concentrations, and performed wet to dry ratio.

### Quantitative organ culture

To determine the bacterial loads in the organs and blood, tissue homogenates or blood were loaded on 5% blood agar plates (Teknova; Hollister, California, USA) after surgical dilutions and incubated for 18 hours as previously described (15). Colonies of all morphologies on plates were counted. For neutrophil depletion experiment, 250 µg of rat anti-Ly6G antibody (clone 1A8, Bio X Cell, Lebanon, NH) was injected intraperitoneally one day before CLP procedure as previously described (16). As a control, normal rat IgG2a isotype control was used.

### *Escherichia coli* intraabdominal infection model

Escherichia coli (*E. coli*)-GFP (ATCC25922-GFP) was overnight cultured in Luria-Bertani (17) ampicillin (100 µg/mL) medium at 37°C and washed twice with sterile PBS buffer. Mice were subjected to an intraperitoneal injection of *E. coli*- GFP (10^8^ CFU). At 6 hours after the injection, peritoneal fluid was collected by lavage using 5 mL of cold PBS buffer. Serial dilutions of the final bacterial inocula were plated on LB-ampicillin (100 µg/mL) agar plates and incubated overnight at 37°C to verify the number of live bacteria injected as above.

### Cell death assay

Cell death was examined using Annexin V apoptosis detection kit (BD Biosciences, San Jose, CA, USA). Briefly cells were stained with Annexin V-FITC. Then, cells were subjected to flow cytometry analysis using BD Accuri C6 (BD Biosciences).

### Chimeric mouse experiments

To generate mixed bone marrow chimeras, recipient mice on the C57BL/6 background were irradiated with two doses of 550 rad with 4-hour intervals. Wild-type (WT; CD45.1) and CD11dKO (CD45.2) derived bone marrow cells (total of 5 × 10^6^ cells) were mixed at the ratio of 1:1 and injected into the tail vein of lethally irradiated recipients. Mice were evaluated for the reconstitution of the immune compartment at various time points after bone marrow transplantation. To prevent bacterial infection, the mice were provided with autoclaved drinking water containing sulfatrim for 1 week prior to and for 4 weeks after irradiation.

### Phagocytosis

The neutrophil phagocytosis was done using Phagotest Kit (Glycotope Biotechnology; Heidelberg, Germany). Mouse neutrophils were incubated in complete RPMI 1640 on ice for 10 min, followed by the addition of FITC-*E. Coli*. Then the neutrophil suspension was kept on ice as cold control or was put into 37°C water bath for 10 min. At the end of incubation, cells were transferred back on ice, quenched, and washed. Cells were suspended in PBS/1% PFA and measured by BD Accuri C6 (BD Biosciences).

### Reactive oxygen species

Mouse neutrophils (2 × 10^5^ in 200 µl) were cultured in complete RPMI 1640 at 37°C for 30 min. Dihydrorhodamine-123 (1 µM; Sigma-Aldrich) was added for 5 min at 37°C. Neutrophils were washed once. PMA (100 nM) was added, and the cells were incubated for additional 15 min at 37°C. After one wash, the cells were resuspended in cold PBS with 1% FCS for detection of ROS-induced rhodamine-123 on BD Accuri C6 (BD Biosciences).

### Chemotaxis

Bone marrow neutrophils were subjected to horizontal chemotaxis assay using the EZ-TAXIScan apparatus (Effector Cell Institute; Tokyo, Japan) as previously performed. Neutrophils suspended in RPMI1640 containing 10 mM HEPES, 0.1% BSA and 10 mM EDTA were aligned on one edge of the chemotaxis channel. At the other end, 1 µM N-formylmethionine-leucyl-phenylalanine (fMLP) was injected, creating a gradient along the channel. Pictures were taken every 30 seconds for 20min, and recorded movies were analyzed using FIJI software (National Institute of Health; Bethesda, MD).

### Eicosanoid lipidomics

Reverse-phase mass spectrometry (MS)-based quantitation technique for eicosanoids was previously described (18). The lipids were extracted with methanol and diluted with water containing 0.1% formic acid to yield a final methanol concentration of 20%. After addition of deuterium-labeled internal standards, the samples were loaded on Oasis HLB cartridge (Waters, Milford, MA). The column was washed with 1 mL of water, 1 mL of 15% methanol, and 1 mL of petroleum ether and then eluted with 0.2 mL of methanol containing 0.1% formic acid. Eicosanoids were quantified by reverse-phase-HPLC-electrospray ionization-tandem MS method.

### RNA sequencing

Blood or lung cells were stained with anti-Ly6G, CD11b, and CD45.2 antibodies. Neutrophils were sorted as Ly6G^+^/CD11b^+^/CD45.2^+^ population. They were subjected to RNA purification using Qiagen RNeasy Plus Mini Kit. RNA samples were quantified using Qubit 2 Fluorometer (Life Technology; Carlsbad, CA) and RNA integrity was checked with Agilent TapeStation (Agilent Technologies; Palo Alto, CA). SMART-Seq v4 Ultra Low Input kit for Sequencing was used for full-length cDNA synthesis and amplification (Clonetech; Mountain View, CA), and Illumina Nextera XT library was used for sequencing library preparation. Briefly, cDNA was fragmented, and adaptor was added using transposase, followed by limited-cycle PCR to enrich and add index to the cDNA fragments. The final library was assessed with Qubit 2.0 Fluorometer and Agilent TapeStation. The sequencing libraries were multiplexed and clustered on one lane of a flowcell. After clustering, the flowcell was loaded on the Illumina HiSeq instrument according to the manufacturer’s instructions. The samples were sequenced using a 2x150 paired end configuration. After investigating the quality of the raw data, sequencing reads were trimmed to remove possible adapter sequences and nucleotides with poor quality using Trimmomatic v.0.36. The trimmed reads were mapped to the mouse reference genome using STAR aligner v.2.5.2b. Only unique reads that fell within exon regions were counted. After extraction of gene hit counts, the gene hit counts table was used. Using DESeq2, a comparison between the groups of samples was performed. The Wald test was used to generate p-value and Log_2_ fold changes. Genes with adjusted p-values < 0.05 and absolute log_2_ fold change > 1 were called differentially expressed genes. Data are available at GSE215749 (GEO).

### CRISPR/Cas9 deletion of Itgad gene in HL-60 cells

CRISPR/Cas9 editing to delete αD (gene: Itgad) expression was done in HL-60 cells as follows. HL-60 cells were cultured at 37°C in RPMI1640 supplemented with 10% FBS, 1% penicillin/streptomycin. Confluency was maintained between 3 x10^5^-1.5x 10^6^/ml. Electroporation was performed one day after passaging when cells were in log phase of growth using the Lonza 4D Nucleofector with 20 µl Nucleocuvette strips as described (19, 20). Briefly, ribonucleoprotein (RNP) complex was made by combining 100 pmol Cas9 (IDT; Newark, NJ) and 100pmol modified sgRNA (Synthego; Redwood City, CA) targeting ITGAD using either sg1 (UUCUUAUCAUGGAUUCAACC) or sg2 (AUGAUAAGAAGCCAGGACUG) and incubating at room temperature for 15 minutes.

2 x10^5^- 4 x 10^5^ HL-60 cells were resuspended in 20 µl SF cell line solution (Lonza; Basel, Switzerland) and were mixed with RNP and underwent nucleofection with program EN-138 as per manufacturer recommendations. Cells were returned to RPMI1640 media and editing efficiency was measured 48 hours after electroporation by polymerase chain reaction (PCR). First, genomic DNA was extracted using the DNeasy kit (Qiagen; Hildgen, Germany) according to the manufacturer’s instructions. Genomic PCR was performed using Platinum II Hotstart Mastermix (Thermo Fischer Scientific), and edited allele frequency was detected by Sanger sequencing and analyzed by ICE (21). The following primer pairs were used: ITGAD (forward: ATGATAAGAAGCCAGGACTG; reverse: CAGTCCTGGCTTCTTATCAT).

### Statistical analysis

Data were analyzed as indicated in the figure legends. Statistical analysis methods were included under each figure legend. Statistical significance was defined as P<0.05. All the statistical calculations were performed using PRISM9 software (GraphPad Software, La Jolla, CA).

## Results

### αDβ2 deficiency attenuated lung injury and improved survival in experimental polymicrobial abdominal sepsis

**Figure 1:**
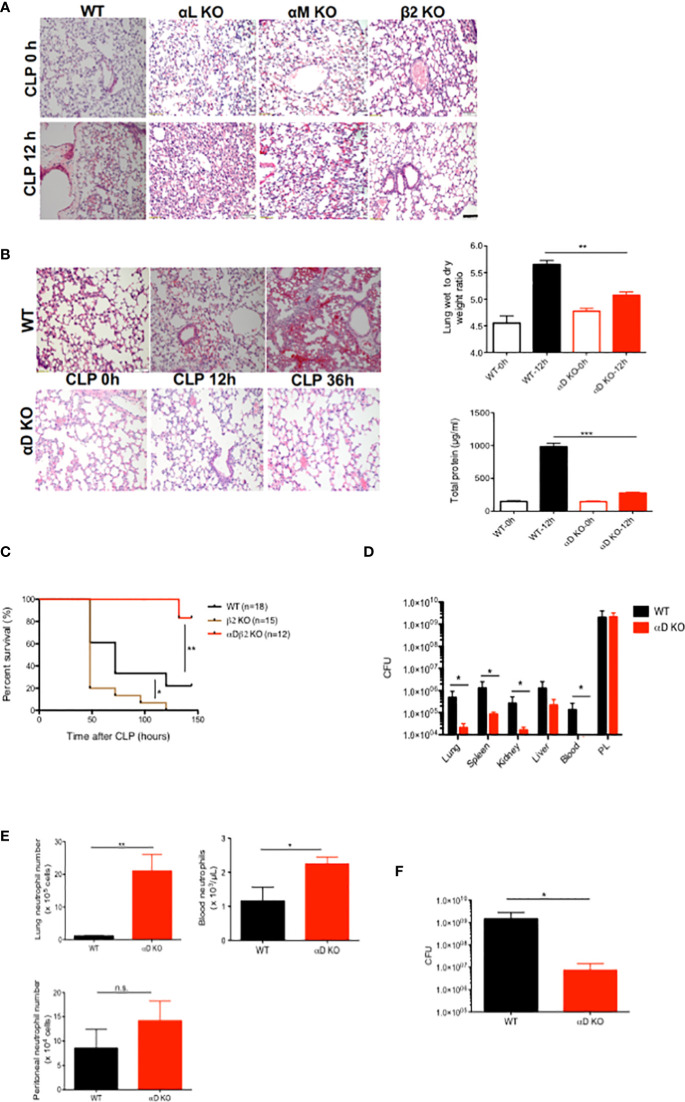
The role of αDβ2 in sepsis. **(A)** Representative lung histology of WT, αL KO, α KO, and β2 KO mice at the baseline and at 12 hours after CLP. Black bar indicates 50 µm. **(B)** Representative lung histology of WT and αD KO at the baseline, at 12 and 36 hours after CLP. Wet to lung analysis and BAL total protein analysis at the baseline and at 12 hours after CLP. Data were shown as mean +/- S.D. of 6 mice per group. One-way ANOVA with Bonferroni *post hoc* analysis was performed. **p < 0.01, ***p < 0.001. **(C)** Survival after CLP in WT (n=18), αD KO (n=12) and β2 KO (n=15) mice. Cox regression analysis was performed. *p <0.05, **p <0.01. **(D)** Bacterial loads in WT and αD KO mice at 12 hours after CLP. Data were shown as mean +/- S.D. of 8 mice per group. Two-way ANOVA analysis was performed. *p < 0.05. **(E)** Neutrophil counts at the lungs, the blood, and the peritoneal cavity in WT and αD KO mice at 12 hours after CLP. Data were shown as mean +/- S.D. of 6 mice. Student t test was performed. *P < 0.05, p < 0.01. **(F)** Bacterial loads at the peritoneal cavity at 6 hours after *E coli* intraperitoneal injection. Data were shown as mean +/- S.D. of 6 mice. Student t test was performed. *p < 0.05.

Polymicrobial abdominal sepsis induced by CLP surgery is the most frequently used preclinical sepsis model that best recapitulates human sepsis (22, 23). We previously reported that sepsis outcomes of αL KO, αM KO and αX KO mice were worse than that of wild-type (11) mice in this model (5–7). Because blocking β2 integrin as a whole attenuated lung injury (9, 10), we examined the degree of lung injury in mice deficient of each of the β2 integrin members. In line, β2 KO mice had less lung injury following CLP (**Figure 1A**). Histological analysis demonstrated that lung injury was highly induced following sepsis in WT, αL KO, and αM KO mice (**Figure 1A**). In contrast, αD KO mice manifested less histological lung injury (**Figure 1B**). Wet-to-dry and BAL fluid analyses also supported the finding (**Figure 1B**). αD KO mice also showed better survival compared to WT mice (**Figure 1C**), suggesting that αDβ2 may be a good target to attenuate sepsis. We further examined bacterial loads at various organs. Bacterial loads at the lungs, spleen, kidney, and blood were significantly less in αD KO mice (**Figure 1D**), consistent with the survival data. However, bacterial loads at the peritoneal cavity did not show any difference between the two strains (**Figure 1D**). Neutrophils are the first-line defense immune cells critical for bacterial eradication (24). The relationship between neutrophil numbers in the blood, the peritoneal cavity, and the lungs between the two strains correlated with the data of bacterial loads above (**Figure 1E**), suggesting that the difference in neutrophil numbers at various sites was in part responsible for the degree of bacterial clearance. CLP sepsis is a sepsis with mixed bacterial flora. Considering the possibility that the flora between the two strains would not likely be the same., we also examined bacterial clearance in *E. coli* intraperitoneal injection model. Bacterial loads were significantly less in αD KO mice at 6 hours after an intraperitoneal injection (**Figure 1D**), indicating that integrin αD deficiency might enhance bacterial eradication.

### αDβ2 KO neutrophils helped to eradicate bacterial loads in the lungs preferentially

**Figure 2:**
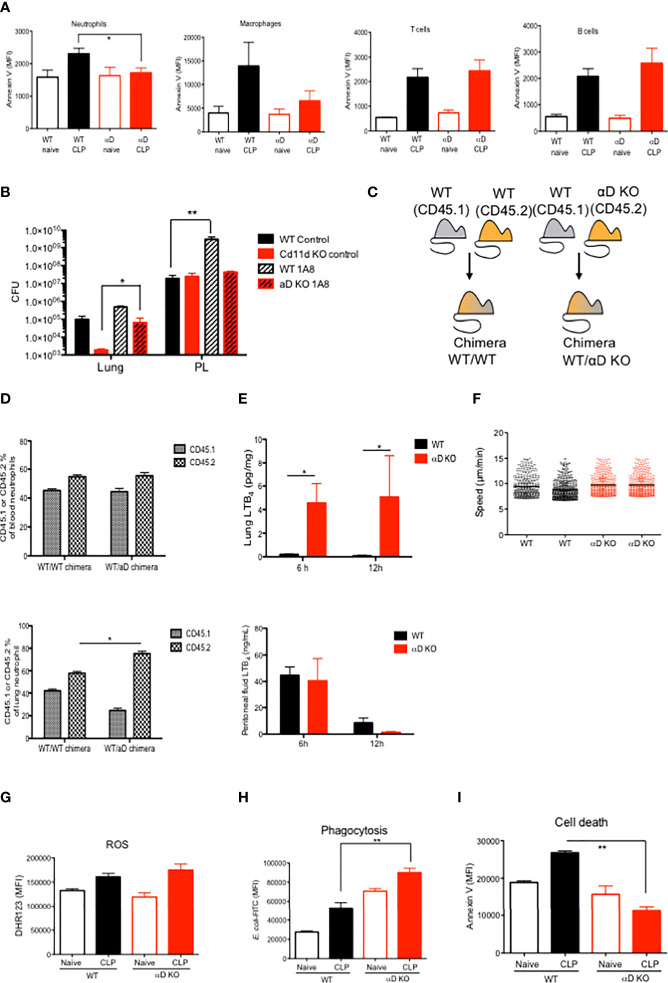
The functional of αDβ2 in neutrophils. **(A)** WT and αD KO lung leukocyte Annexin V expression at the baseline and at 12 hours after CLP. Data were shown as mean +/- S.D. of 4 mice. MFI; mean fluorescence intensity. One-way ANOVA with Bonferroni *post hoc* analysis was performed. *p < 0.05. **(B)** Bacterial loads at the lungs and the peritoneal lavage in WT and αD KO mice with or without neutrophil depletion. Mice were subjected to CLP sepsis and the samples were obtained at 12 hours after CLP. Data were shown as mean +/- S.D. of 6 mice. One-way ANOVA with Bonferroni *post hoc* analysis was performed. *p < 0.05, **p < 0.01. **(C)** Chimera scheme. **(D)** Blood and lung neutrophil analysis of chimeric mice at 12 hours after CLP. Data were shown as mean +/- S.D. of 4 mice. One-way ANOVA with Bonferroni *post hoc* analysis was performed. *p < 0.05. **(E)** Lung and peritoneal cavity LTB_4_ levels at 6 and 12 hours after CLP. Data were shown as mean +/- S.D. of 4 mice. One-way ANOVA with Bonferroni *post hoc* analysis was performed. *p < 0.05. **(F)** LTB_4_ mediated bone marrow neutrophil chemotaxis assay. Each dot represents one neutrophil. One-way ANOVA with Bonferroni *post hoc* analysis was performed. No significance was observed. **(G)** Lung neutrophil ROS activity from naïve and post CLP mice. For CLP mice, lungs were obtained at 12 hours after CLP. Data were shown as mean +/- S.D. of 6 mice. One-way ANOVA with Bonferroni *post hoc* analysis was performed. No significance was observed. **(H)** Lung neutrophil phagocytosis activity from naïve and post CLP mice. For CLP mice, lungs were obtained at 12 hours after CLP. Data were shown as mean +/- S.D. of 6 mice. One-way ANOVA with Bonferroni *post hoc* analysis was performed. **p < 0.01. **(I)** Lung neutrophil Annexin V expression from naïve and post CLP mice. For CLP mice, lungs were obtained at 12 hours after CLP. Data were shown as mean +/- S.D. of 6 mice. One-way ANOVA with Bonferroni *post hoc* analysis was performed. **p < 0.01.

The correlation of the degree of splenic apoptosis with sepsis outcome has been previously described (25–27). Splenic neutrophil and monocyte cell death was significantly less in αD KO mice (**Figure 2A**), consistent with the sepsis outcome data above. However, the degree of cell death was comparable in T and B cells between WT and αD KO mice (**Figure 2A**). The expression of αDβ2 was previously reported on neutrophils and monocytes/macrophages (28). We examined the contribution of neutrophils to CLP sepsis in both WT and αD KO mice by depleting neutrophils using 1A8 antibody. Neutrophil depletion worsened both lung and peritoneal bacterial loads in WT mice, but it significantly worsened only lung bacterial loads in αD KO mice (**Figure 2B**), which would likely be due to the difference in lung bacterial loads after CLP between the two strains. While lung bacterial loads were significantly lower in αD KO mice receiving isotype control compared to WT mice receiving isotype control, they were comparable between WT and αD KO mice subjected to neutrophil depletion.

### αD KO neutrophil demonstrated higher phagocytic ability, less apoptosis and more tissue recruitment

Although we observed the difference in neutrophil numbers in the lungs between WT and αD KO mice, it is yet to be determined if the result was intrinsically derived from integrin αD. To validate if the result of higher αD KO neutrophil numbers in the lungs is intrinsic, we generated mixed bone marrow chimera to harbor both WT and αD KO mice-derived hematopoietic systems, which were distinguished by congenic markers CD45.1 and CD45.2, respectively. Control mixed chimeras were made by the reconstitution of CD45.1 and CD45.2 WT bone marrow cells (**Figure 2C**). At 12 weeks after bone marrow transplantation, we examined peripheral blood and confirmed the peripheral blood leukocytes were constituted equally by CD45.1 and CD45.2 cells (data not shown). We found that more αD KO derived neutrophils existed in the lungs compared to WT (CD45.1) neutrophils, supporting that the phenotype was intrinsic to αD KO neutrophils (**Figure 2D**). LTB_4_ is a major neutrophil chemoattractant. At 6 and 12 hours after CLP in WT and αD KO mice, we observed LTB_4_ level was significantly higher in the lungs of αD KO mice (**Figure 2E**). In contrast, peritoneal LTB_4_ level was not statistically significant between the strains. We did not see any difference in neutrophil recruitment to neutrophil chemoattractant LTB_4_ *in vitro* (**Figure 2F**), indicating that the difference of numbers between WT and αD KO neutrophils in chimera’ lungs was not driven by chemotaxis. Here we examined the role of αDβ2 in neutrophils using WT and αD KO neutrophils. The level of reactive oxygen species (ROS) production was comparable between WT and αD KO mice (**Figure 2G**), while there was an increased phagocytosis by lung αD KO neutrophils (**Figure 2H**), supporting the result of the *E. coli* injection model (**Figure 1F**). To examine if higher αD KO neutrophil counts in the lung is explained by neutrophil survival, we also examined the cell death of lung neutrophils. Consistent with the result of splenic neutrophil apoptosis, the cell death of αD KO lung neutrophils was less compared to WT neutrophils (**Figure 2I**), which at least in part explains these results. Taken together, αD KO mice demonstrated a difference in neutrophil phenotypes characterized as less cell death and more efficient phagocytosis in the lungs. Higher lung αD KO neutrophil counts will be likely in part responsible for less neutrophil cell death.

### αD KO neutrophil transcriptomic analysis demonstrated the association between αD and proliferation, and phagocytosis

**Figure 3:**
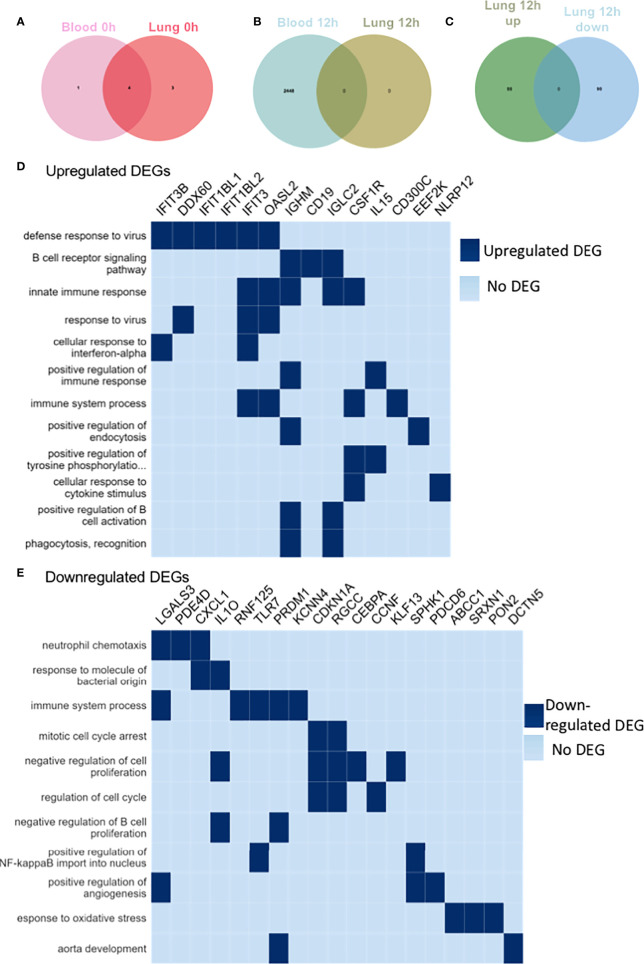
RNA sequencing of blood and lung neutrophils following CLP sepsis. RNA seq of triplicates for blood and lung neutrophils at baseline and 12 hours after CLP. Neutrophils were sorted by CD45^+^Ly6G^+^CD11b^+^ population. **(A)** Venn diagram showing significant DEGs in the blood and lung of αD KO mice at 0h post-CLP compared to WT mice at 0h post-CLP. The light pink circle represents DEGs in the blood of αD KO mice at 0h post-CLP compared to the blood of WT mice at 0h post-CLP. The deep pink circle represents DEGs in the lung of αD KO mice at 0h post-CLP compared to the lung of WT mice at 0h post-CLP. The intersection of the two circles represents overlapping DEGs in the blood and lung of αD KO mice at 0h post-CLP compared to WT mice at 0h post-CLP. Overall, only 5 genes were differentially expressed in the blood of αD KO and 7 genes in the lungs of αD KO compared to the WT mice at the baseline (0h post-CLP) and 4 of these DEGs were common in the blood and lung, suggesting no significant differences in the transcriptomic profile of WT and αD KO mice at the baseline. **(B)** Venn diagram showing significant DEGs in the blood and lung of αD KO mice at 12h post-CLP compared to αD KO mice at 0h post-CLP. The light blue circle represents DEGs in the blood of αD KO mice at 12h post-CLP compared to the blood of αD KO mice at 0h post-CLP. The green circle represents DEGs in the lung of αD KO mice at 12h post-CLP compared to the lung of αD KO mice at 0h post-CLP. The intersection of the two circles represents overlapping DEGs in the blood and lung of αD KO mice at 12h post-CLP compared to αD KO mice at 0h post-CLP. Overall, 2448 genes were differentially expressed in the blood of αD KO at 12h post-CLP compared to 0h post-CLP and none was observed in the lungs, suggesting no differences in the transcriptomic profile of the lungs of αD KO mice between 0h and 12h post-CLP. **(C)** Venn diagram showing significant DEGs in the lung of αD KO mice at 12h post-CLP compared to WT mice at 12h post-CLP. The green circle represents DEGs upregulated in the lung of αD KO mice at 12h post-CLP compared to the lung of WT mice at 12h post-CLP. The blue circle represents DEGs downregulated in the lung of αD KO mice at 12h post-CLP compared to the lung of WT mice at 12h post-CLP. The intersection of the two circles represents overlapping DEGs in lung of αD KO mice at 12h post-CLP compared to WT mice at 12h post-CLP. Overall, 55 genes were significantly upregulated and 90 were significantly downregulated in the lung of αD KO at 12h post-CLP compared to WT mice at 12h post-CLP and these DEGs were used for pathway enrichment analysis **(D, E)**. **(D)** Pathway Enrichment Analysis (GO) of upregulated DEGs in the lung of αD KO mice at 12h post-CLP compared to WT mice at 12h post-CLP. Genes with adjusted p-values < 0.05 and absolute log2 fold change > 1 were considered differentially expressed genes. Heat map was created by plotting the enriched pathways and the genes that have contributed to the enrichment signal (indicated in deep blue). **(E)** Analysis of downregulated DEG pathway in lung of αD KO mice at 12h post-CLP compared to WT mice at 12h post-CLP. Genes with adjusted p-values < 0.05 and absolute log2 fold change > 1 were called differentially expressed genes.

To further characterize αD KO neutrophils, we performed transcriptomic analysis of blood and lung neutrophils. We found only 5 DEGs in blood and 7 DEGs in lung between WT and αD KO mice at the baseline. One DEG was overlapped between blood and lung. This result suggested that the transcriptomic signature of WT and αD KO neutrophils in the blood and lung at the baseline was quite similar (**Figure 3A**). The DEG analysis of αD KO neutrophils at 12 hours post CLP vs at the baseline (time 0h) in blood and lung showed that there was no DEG in lung, suggesting that there was no DEGs in the lung between baseline and 12 hours post-CLP (**Figure 3B**). When we compared lung neutrophils between WT and αD KO mice, we identified 55 upregulated DEGs and 90 upregulated DEGs in αD KO lung neutrophils (**Figure 3C**). Upregulated genes include phagocytosis related genes (**Figure 3D**), and downregulated genes include cell arrest and proliferation genes (**Figure 3E**).

### Human validation

**Figure 4:**
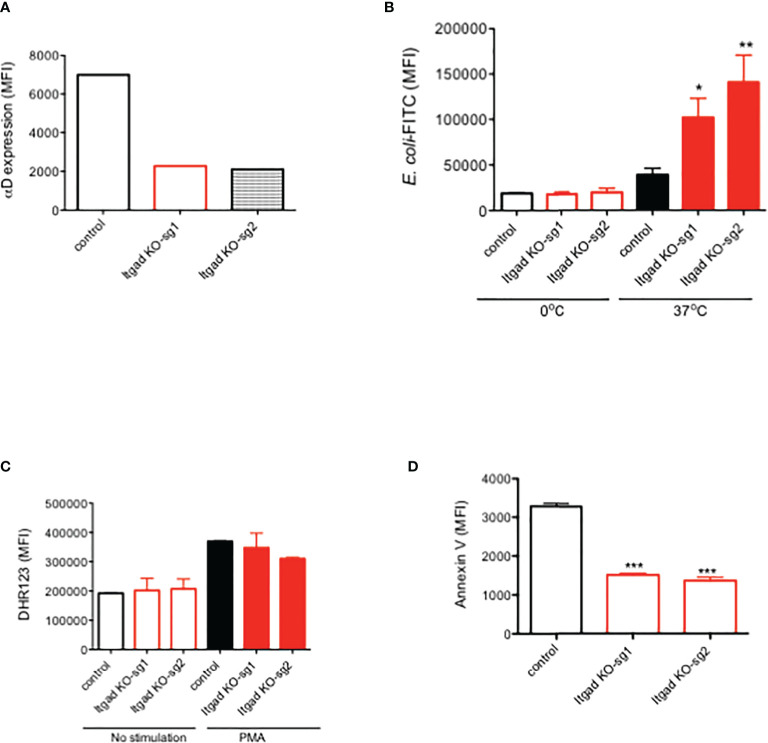
The role of αDβ2 in human HL60 cells. **(A)** αD expression of HL60 cells with or without αD CRISPR/Cas9 deletion. Representative data was shown. **(B)** Phagocytosis, **(C)** ROS, and **(D)** Annexin V expression of HL60 cells with or without αD CRISPR/Cas9 deletion. Data were shown as mean +/- S.D. of triplicates. Sg1 and sg2 denote two different guide RNA. One-way ANOVA with Bonferroni *post hoc* analysis was performed. *P < 0.05, **p < 0.01, ***p < 0.001.

Human neutrophils were previously reported to express αDβ2 (29). To determine the role of αDβ2 in human neutrophils, we performed CRISPR/Cas9 deletion of αD in HL-60 cells. Expression of αD was confirmed by flow cytometry (**Figure 4A**).

Phagocytosis, ROS, and apoptosis were examined. We found that phagocytosis was significantly enhanced in αD KO HL-60 cells compared to the control (**Figure 4B**). No difference in ROS was observed (**Figure 4C**). Cell death was attenuated in αD KO HL-60 cells (**Figure 4D**). This is consistent with the findings in murine experiment, indicating the relevance of our findings in human.

## Discussion

Integrin αDβ2 was the last β2 integrin member cloned (30). β2 integrins work by binding to their ligands. Ligands for αLβ2 include intercellular adhesion molecule (ICAM)-1, -2, and -3, while αMβ2 and αXβ2 bind to iC3b, ICAM-1, and fibrinogen. So far αDβ2 reportedly binds to ICAM-3, VCAM-1, and 2-(ω-carboxyethyl)pyrrole (CEP) (31). Those ligands have been reported from *in vitro* experiments, but how these ligand-αDβ2 interactions play a role *in vivo* has not been largely studied. αDβ2 expression on macrophages (30) and neutrophils (29, 32) has been reported. Foam cells are a type of macrophages that localize to fatty deposits on blood vessels. αDβ2 deficiency was associated with less lipid deposition in the atherosclerosis model (28). The involvement of αDβ2 in spinal cord injury was also described in the context of neutrophil invasion. The administration of anti-αD antibody attenuated spinal cord injury in rats (32, 33). In the context of infection, de Azevedo-Quintanilha et al. reported that αD KO mice demonstrated less lung injury in Malaria infection model (34). Here we reported a novel role of αDβ2 in sepsis model. The deficiency of αDβ2 attenuated lung injury and sepsis outcome, which is significantly important given that a specific treatment is urgently needed for sepsis.

For the first time, we showed that the deficiency of αDβ2 led to 1) the enhancement of phagocytosis and 2) the attenuation of cell death in neutrophils. This finding is interesting in contrast to αMβ2. αMβ2 is also called complement receptor 3 (CR3), and serves as one of the major phagocytosis receptors by binding to iC3b. αMβ2 also affects apoptosis. The deficiency of αMβ2 leads to the defect in apoptosis in the setting of infection (13). The mechanism of apoptosis induction *via* αMβ2 is not completely delineated yet, but it is proposed that phagocytosis of iC3b coated microbes induces ROS production, which leads to apoptosis (35). The major phagocytosis receptors include Fcγ receptor and αMβ2. At this point, it is unclear how the deficiency of αDβ2 leads to an enhancement of phagocytosis. Given that αMβ2 and αDβ2 have high sequence homology, αDβ2 could compete iC3b coated microbes against αMβ2. However, iC3b has not been reported as a ligand for αDβ2. In addition, the attenuation of apoptosis by αDβ2 is also confusing. If αDβ2 deficiency increases phagocytosis, ROS may increase, leading to more apoptosis based on the theory of αMβ2-mediated apoptosis.

The HL-60 cell experiment showed that the effect of αDβ2 on cell death is cell intrinsic. Thus, it is interesting how αDβ2 plays a functional role within neutrophils. Although αLβ2 and αMβ2 bind to ligands outside their cells for their activity, we previously reported that αXβ2 has its own ligand within neutrophils. It may be possible that αDβ2 has its intrinsic ligand within the cell, or surrounding HL-60 cells. For example, ICAM-3 is expressed on neutrophils, serving as a ligand for αDβ2. ICAM-3 binding to neutrophils induces apoptosis (36), so the deficiency of αDβ2 may lessen this interaction, leading to less apoptosis.

From sepsis standpoint, lung injury is one of the most serious organ injuries that patients succumbed to. The mortality of patients with lung injury in sepsis is quite high. Thus, our finding will offer a very interesting target for sepsis therapeutic. So far there is no αDβ2 antagonist reported. We primarily focused on the role of αDβ2 in sepsis in this study. It is also interesting to examine the role of this molecule in other disease states such as cancers. Among β2 integrin members, αMβ2 has been most extensively studied in the context of cancer. Its inhibition enhanced tumor response to radiation (37). Its deficiency suppressed intestinal tumor growth (38). However, its agonist LA1 was reported to attenuate tumor growth (39). Thus, the role of αMβ2 in cancer may not be straightforward. So far there is a limited data available on the role of αDβ2 in cancer, which needs future study.

In conclusion, we found a novel role of αDβ2 in neutrophils and sepsis. Delineating the underlying mechanism of αDβ2-mediated modulation of neutrophil functions will help us to understand how αDβ2 contributes to sepsis pathophysiology for considering as a future therapeutic target.

## Data availability statement

The data presented in the study are deposited in the Gene Expression Omnibus (GEO) repository accession number GSE215749. 

## Ethics statement

The animal study was reviewed and approved by Boston Children’s Hospital IACUC.

## Author contributions

SK - Designed experiment, performed experiment and wrote manuscript. LH - Designed experiment and performed experiment. TO - Designed experiment and performed experiment MS-F - Designed experiment and performed experiment. HL - Designed experiment KY - Designed experiment, performed experiment and wrote manuscript. All authors contributed to the article and approved the submitted version.

## Funding

This study was in part supported by R21HD099194 (KY, SK).

## Conflict of interest

The authors declare that the research was conducted in the absence of any commercial or financial relationships that could be construed as a potential conflict of interest.

## Publisher’s note

All claims expressed in this article are solely those of the authors and do not necessarily represent those of their affiliated organizations, or those of the publisher, the editors and the reviewers. Any product that may be evaluated in this article, or claim that may be made by its manufacturer, is not guaranteed or endorsed by the publisher.
